# Inhibition of VEGFR2 and EGFR signaling cooperatively suppresses the proliferation of oral squamous cell carcinoma

**DOI:** 10.1002/cam4.6282

**Published:** 2023-06-21

**Authors:** Maho Onda, Akinobu Ota, Kunihiro Ito, Takayuki Ono, Sivasundaram Karnan, Mikako Kato, Sayuri Kondo, Akifumi Furuhashi, Tomio Hayashi, Yoshitaka Hosokawa, Yoshiaki Kazaoka

**Affiliations:** ^1^ Department of Oral and Maxillofacial Surgery Aichi Medical University Hospital Nagakute Japan; ^2^ Department of Biochemistry Aichi Medical University School of Medicine Nagakute Japan; ^3^ Department of Food and Nutritional Environment College of Human Life and Environment Kinjo Gakuin University Nagoya Japan

**Keywords:** drug sensitivity, EGFR, genome editing, OSCC, VEGFR

## Abstract

**Background:**

Epidermal growth factor receptor (EGFR) is frequently overexpressed in oral squamous cell carcinoma (OSCC), and EGFR‐targeting therapeutics have been widely employed to treat patients with a variety of carcinomas including OSCC. Here, we aimed to investigate alternative signaling for OSCC survival under the disruption of EGFR signaling.

**Methods:**

OSCC cell lines, namely HSC‐3 and SAS, were utilized to investigate how EGFR disruption affects cell proliferation. Gene set enrichment analysis was performed to examine how EGFR disruption affects oncogenic signaling in OSCC cells. Disruption of *KDR* gene was performed using CRISPR/Cas9 techniques. A VEGFR inhibitor, vatalanib was used to research the impact of VEGFR inhibition on OSCC survival.

**Results:**

EGFR disruption significantly decreased the proliferation and oncogenic signaling including Myc and PI3K‐Akt, in OSCC cells. Chemical library screening assays revealed that VEGFR inhibitors continued to inhibit the proliferation of *EGFR*‐deficient OSCC cells. In addition, CRISPR‐mediated disruption of *KDR*/*VEGFR2* retarded OSCC cell proliferation. Furthermore, combined erlotinib–vatalanib treatment exhibited a more potent anti‐proliferative effect on OSCC cells, compared to either monotherapy. The combined therapy effectively suppressed the phosphorylation levels of Akt but not p44/42.

**Conclusion:**

VEGFR‐mediated signaling would be an alternative signaling pathway for the survival of OSCC cells under the disruption of EGFR signaling. These results highlight the clinical application of VEGFR inhibitors in the development of multi‐molecular‐targeted therapeutics against OSCC.

## Graphical abstract

Despite the pivotal role of epidermal growth factor receptor (EGFR) in cancer, little is known about the alternative cellular signaling for the proliferation and/or survival in the absence of EGFR. This is the first study to demonstrate the role of KDR/VEGFR2 on the survival of oral squamous cell carcinoma. VEGFR2 inhibitor vatalanib and erlotinib cooperatively reduced the phosphorylation levels of Akt.

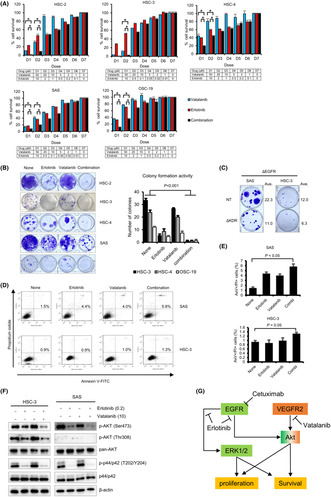

## 1 INTRODUCTION

Receptor tyrosine kinases are transmembrane receptors, which serve as molecular targets for clinical applications due to their pivotal role in tumor development. Epidermal growth factor receptor (EGFR), which is encoded by the proto‐oncogene *c‐ErbB‐1*, is normally expressed in the epidermis at the physiological level and is frequently upregulated or sporadically mutated in various types of solid cancers including non‐small‐cell lung cancer (NSCLC), colorectal, breast, pancreatic, and head and neck squamous cell carcinoma (HNSCC).^1^, ^2^, ^3^ Through the activation of various intracellular signaling pathways such as RAS‐MAPK, PI3K‐Akt, and cyclin‐dependent kinases (CDKs), EGFR overexpression and mutations (Most are deletions in exon 19 or L858R in exon 21) confer a dysregulated and/or constitutive proliferative activity to cancer cells. Overexpression of EGFR has been detected in more than 27.9% of OSCC cases and is associated with lymph node metastasis.^4^ Based on the molecular pathogenesis, the strategies to directly inhibit EGFR activity with small chemicals (e.g., gefitinib) and with biopharmaceuticals (e.g., cetuximab) should have worked as promising molecular‐targeted therapies paired with other chemotherapeutics and radiation therapy in cancer treatment.^5^, ^6^, ^7^, ^8^, ^9^, ^10^ In fact, cetuximab monotherapy and/or combined therapies often bring favorable responses toward patients.^11^, ^12^, ^13^, ^14^, ^15^ However, the prognosis is still relatively unfavorable, with the 5‐year overall survival estimated to be less than 50% in OSCC.^16^, ^17^

Approximately two decades have passed since researchers began investigating the limited anti‐cancer effect of EGFR‐targeted therapeutics in cancer. In lung cancer, acquired resistance following treatment with EGFR‐targeted therapies has been thoroughly examined using patient samples, in which additional gene mutations and/or hyper‐amplification in EGFR (e.g., T790M in EGFR) as well as downstream genes including MET, *c‐ErbB‐2*/HER2, RAS/MAPK, and cyclins/CDKs were identified.^18^ Clarification of vital molecules in cancer cells exposed to EGFR‐targeted therapeutics will enable us to develop novel therapeutics to overcome such drug resistance. In OSCC, Yang et al reported that the inhibition of hepatocyte growth factor‐MET activity sensitizes OSCC cells to cetuximab.^19^ However, little is known about the molecular mechanism by which OSCC cells escape the antiproliferative effect of EGFR‐targeted drugs. Therefore, it would be of particular interest to discover alternative signaling for the survival of OSCC under EGFR inhibition.

In this study, we generated EGFR‐deficient cell clones in the HSC‐3 and SAS cells and assessed how EGFR deficiency affected cell proliferation. We also demonstrated the gene expression profiling to highlight how the oncogenic signaling pathway is impacted by EGFR deficiency. A chemical drug screening assay was performed to uncover the drug sensitivity of EGFR‐deficient OSCC cells in vitro. The potential molecular mechanism underlying the additive anti‐proliferative efficacy of the combined erlotinib–vatalinib treatment is also discussed.

## 2 MATERIALS AND METHODS

### 2.1 Reagents

Roswell Park Memorial Institute medium (RPMI1640) and Penicillin–streptomycin (P/S) solution were obtained from FUJIFILM‐Wako Chemical Corporation. Trypsin–EDTA in phosphate‐buffered saline was purchased from BioConcept Ltd. Fetal bovine serum (FBS) was purchased from Nichirei Bioscience Inc. An EGFR inhibitor Erlotinib (OSI‐774) HCl and VEGFR inhibitors Lenvatinib (E7080) and vatalanib (PTK787) 2HCl were obtained from Selleck Biotech.

### 2.2 Cell culture

Human OSCC cell lines HSC‐2, HSC‐3, HSC‐4, SAS, and OSC‐19, all of which were purchased from the Japanese Collection of Research Bioresource Cell Bank, were incubated at 37°C in RPMI1640 with 5% heat‐inactivated FBS and P/S in a 5% CO_2_ humidified atmosphere. The cells were seeded into 6‐ or 96‐well plates by dispensing them from the dishes and/or flasks with 0.05% trypsin–EDTA for each experiment.

### 2.3 Gene knockout using CRISPR/Cas9 system

The CRISPR/Cas9 system was used to disrupt the expression of the *EGFR* and *KDR*/*VEGFR2* genes, as described previously.^20^ pSpCas9(BB)‐2A‐GFP (PX458) for *EGFR* gene knockout and lentiCRISPR v2 for *KDR/VEGFR2* gene knockout were gifted by Feng Zhang (plasmid #48138 for PX458 and plasmid #52961 for lentiCRISPR v2; Addgene).^21^, ^22^ A single guide RNA (sgRNA) sequence was selected using E‐CRISP (http://www.e‐crisp.org/E‐CRISP/designcrispr.html). The sgRNA sequences for *EGFR* and *KDR/VEGFR2* are shown as follows: 5’‐TGGGGCCGACAGCTATGAGA for *EGFR* (exon 8) and 5’‐GAAACCTGTCCACTTACCTG for *KDR/VEGFR2* (exon 20). For lentivirus preparation, 293 T cells (4 × 10^6^ cells/dish) were seeded in a 10‐cm dish 1 day before transfection. For EGFR gene knockout, a PX458 vector containing either no sgRNA or *EGFR* sgRNA was introduced into HSC‐3 and SAS cells using 4D Nucleofector (Lonza Japan). After cell sorting with a FACSAria III (BD Biosciences) instrument, a single clone was selected from a 96‐well plate, expanded in a 12‐well plate, and used for biological assays. Lentiviral lentiCRISPR v2 containing *KDR/VEGFR2* sgRNA, viral packaging vector psPAX2 (a gift from Didier Trono; plasmid #12260; Addgene), and viral envelope vector pCMV‐VSV‐G (a gift from Bob Weinberg; plasmid #8454; Addgene) were diluted at 4:3:2 ratio in Opti‐MEM medium (Thermo Fisher Scientific).^23^ After infection, puromycin selection was performed, and the drug‐resistant cells were used for the following biological assays.

### 2.4 Gene expression analysis

The experimental procedure for the cDNA microarray analysis was performed as described previously.^24^ The general methodology used in this study was based on the manufacturer's protocol (Agilent Technologies). In brief, cDNA synthesis and cRNA labeling with the cyanine 3 dye were carried out using the Agilent Low Input Quick Amp Labeling Kit (Agilent Technologies). The cyanine 3‐labeled cRNA was purified, fragmented, and hybridized on a human gene expression 4 × 44 K v2 microarray chip containing 27,958 Entrez Gene RNAs, using a Gene Expression Hybridization kit (Agilent Technologies). The raw and normalized microarray data have been submitted to the GEO database at NCBI (GSE222629). Gene set enrichment analysis was carried out according to the instructions.^24^, ^25^, ^26^

### 2.5 Cell proliferation (MTT) assay

MTT assay was performed as described previously.^27^ Briefly, OSCC cells (1.25 × 10^3^ cells per well) were seeded into a 96‐well culture plate. At the days 0, −2, −4, and −6, MTT reagent was added into each well and the cells were incubated for 4 h at 37°C in a 5% CO_2_ humidified atmosphere followed by overnight incubation with lysis buffer. The absorbance at 595 nm was scanned by a SpectraMAX M5 spectrophotometer (Molecular Devices).

### 2.6 Chemical library screening assay

A custom chemical compounds library was obtained from Selleck Biotech. The library consists of 800 compounds (FDA‐approved, preclinical, natural, kinase‐related, cell cycle–related, angiogenesis, JAK/STAT–related, and transcription‐related compounds). HSC‐2 and SAS cells (1.25 × 10^3^ cells per well) were seeded into a 96‐well plate and cultivated for 48 h. The cells were treated with each compound (5 μM) or DMSO (0.05%) for 72 h. After incubation, MTT assay was performed as described above. Percentages of cell survival were calculated following normalization to the mean optical densities in DMSO‐treated cells, which were arbitrarily defined as 100%. To select chemical compounds which exhibit stronger anti‐proliferative effects on EGFR‐KO cells, differential percentages of cell survival between EGFR1‐KO cells and NT cells were calculated.

### 2.7 Apoptosis assay

Briefly, HSC‐3 and SAS cells were seeded into a 12‐well culture plate (1 × 10^5^ cells per well). On the following day, the cells were treated with 1 μM of Erlotinib and/or 10 μM of VEGFR inhibitors (lenvatinib and vatalanib) for 48 h. After incubation, the cells were dispersed with Accutase (Innovative Cell Technologies, Inc.) and were incubated with annexin V (Ax)‐FITC (Medical & Biological Laboratories Co., Ltd.) and propidium iodide (PI, Calbiochem) at room temperature for 15 min. The cell suspension was subjected to flow cytometry analysis using a FACSCantoII (BD) system. A total of 10,000 cells passing through both forward and side scatters were counted.

### 2.8 Immunoblot analysis

Immunoblot analysis was performed as described previously.^28^ Antibodies against VEGFR2 (A5609, ABclonal), EGFR (#4267, CST), phospho‐p44/p42 (#4370, CST), p44/42 (#4695), phospho‐Akt (T308, #2965, CST), phospho‐Akt (S473, #4060), pan‐Akt (#4691, CST), and β‐Actin (66009‐1‐Ig; Proteintech Japan) were used with a working dilution in tris buffered saline (TBS) containing 0.1% Tween 20 (TBS‐T) and 2% BSA. Immune complexes were detected using ImmunoStar LD (Fujifilm‐Wako Pure Chemical) in conjunction with an Amersham Imager 600 (GE Healthcare).

### 2.9 Combination index (CI) and dose reduction index (DRI) analyses

The multiple drug effect analysis of Chou and Talalay,^29^, ^30^ which is based on a median‐effect principle, was used to calculate combined drug effects. The CI and DRI for determining synergism and antagonism between vatalanib and erlotinib were calculated as described previously.^31^ Synergism, additivity and antagonism are indicated by CI <1, CI = 1, and CI >1, respectively.

### 2.10 Statistical analysis

The data are shown as the average values ± SE (*n* = 3). We defined the statistical significance between groups as *p* < 0.05 using a student's *t*‐test in the study. Statistical analyses were performed with EZR (Saitama Medical Center, Jichi Medical University, Saitama, Japan), which is a graphical user interface for R.^32^

## 3 RESULTS

### 3.1 Disruption of EGFR retards the proliferation of OSCC cells

**FIGURE 1:**
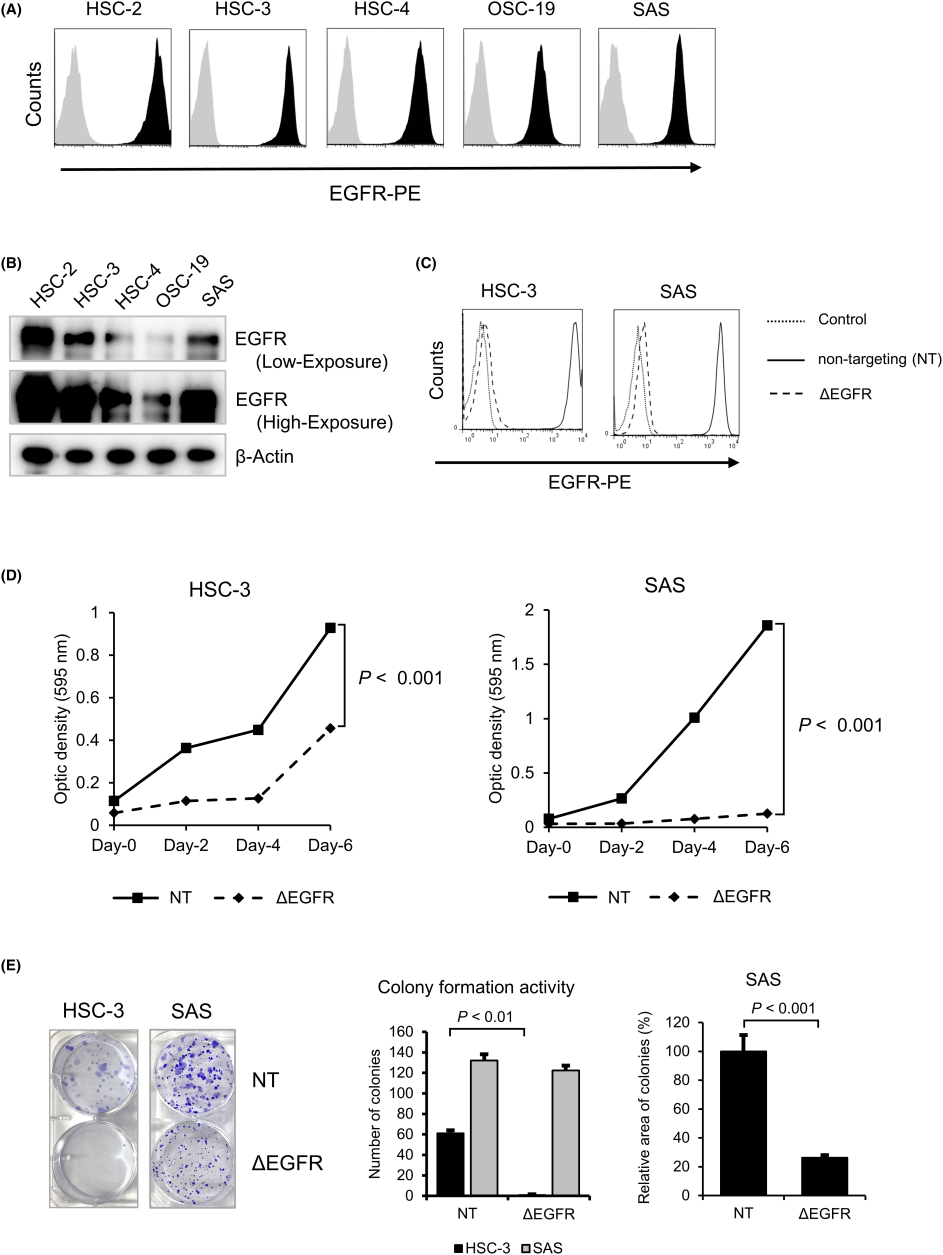
Disruption of epidermal growth factor receptor (EGFR) exhibits growth retardation in oral squamous cell carcinoma (OSCC) cells. (A,B) Expression of EGFR in the OSCC cell lines tested (HSC‐2, HSC‐3, HSC‐4, OSC‐19, and SAS). Flow cytometry (A) and Western blot (B) analyses were performed to detect EGFR expression with specific antibodies against EGFR. (C) CRISPR‐based disruption of EGFR in HSC‐3 and SAS cells was confirmed using FACS analysis with a PE‐conjugated anti‐EGFR antibody. The cell surface expression of EGFR was examined with non‐targeting (NT) and/or EGFR‐targeting (ΔEGFR) in HSC‐3 and SAS cells. Dotted line, stained with control antibody; solid and dashed lines, stained with anti‐EGFR‐PE antibody against NT and EGFR‐KO cells, respectively. (D) OSCC cells (NT/HSC‐3, ΔEGFR/HSC‐3, NT/SAS, and ΔEGFR/SAS) were seeded into 96‐well plates and were incubated for the indicated time (day‐0, day‐2, day‐4, and day‐6). The optic densities at each time points are shown. Solid line, NT cells; Dotted line, ΔEGFR cells. (E) Colony formation assay. The OSCC cells (200 cells/well in a 6‐well plate) were seeded and were incubated for 10 days. The number of colonies (*N* = 3, mean ± SE) and sizes of colonies (*N* = 20, mean ± SE) are shown in right and left panels, respectively.

We first examined the expression of EGFR in the OSCC cell line. Flow cytometry analysis and Western blot analysis revealed that all the OSCC cell lines evaluated exhibited high expression levels of EGFR (Figure 1A,B). We then investigated the impact of EGFR disruption on cell growth using a CRISPR/Cas9 system to elucidate the role of EGFR in the proliferation of OSCC cell lines. EGFR expression were only seen in HSC‐3 and SAS cells, which allowed flow cytometry analysis to demonstrate that CRISPR‐mediated disruption of EGFR had occurred (Figure 1C). In addition, Sanger sequence analysis confirmed the heterozygous disruption of the *EGFR* gene in both *EGFR*‐knockout (KO) HSC‐3 and SAS cell lines (Figure S1). MTT assay revealed that the growth rates in the EGFR‐KO HSC‐3 and SAS cells were significantly reduced when compared with those of the non‐targeting (NT) cells (Figure 1D). Colony formation assay also showed that the number of colonies and/or the size of colonies in the EGFR‐KO cells was lower than that in the NT cells (Figure 1E). These results suggested that EGFR mediates the cell proliferation signaling in OSCC cells.

### 3.2 Disruption of EGFR inactivates oncogenic gene signatures in OSCC cells

**FIGURE 2:**
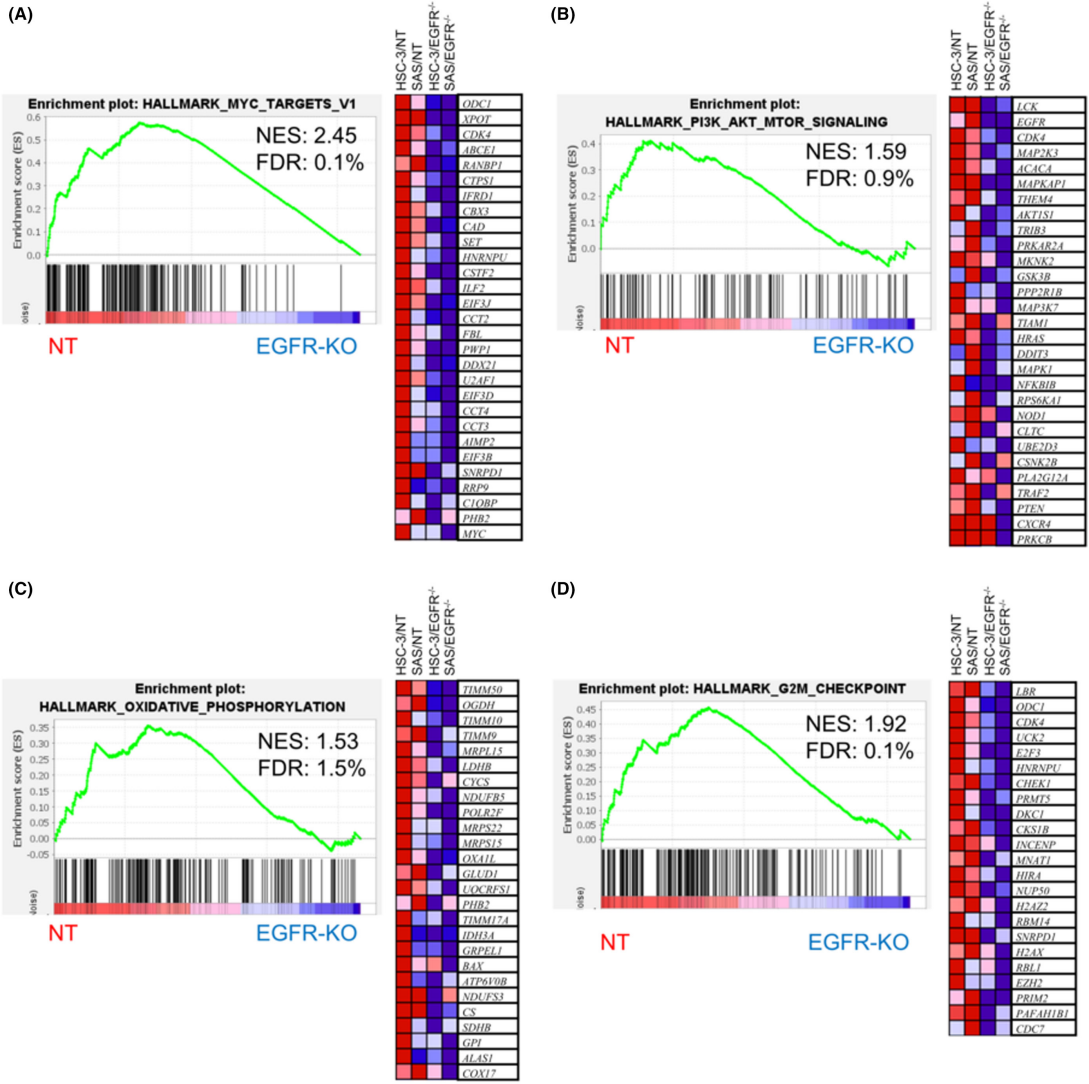
Inactivation of oncogenic gene signatures in epidermal growth factor receptor (EGFR)‐KO oral squamous cell carcinoma (OSCC) cells. GSEA was conducted using the GSEA v4.1.0 software and the Molecular Signatures Database (Broad Institute). All raw data were formatted and applied to hallmark gene sets (h). Representative GSEA enrichment plots and corresponding heatmap images of the indicated gene sets in the EGFR‐KO and non‐targeting (NT) cells are shown. Genes contributing to enrichment are shown in rows, and the sample is shown in one column of the heatmap. The expression level is represented as a gradient from high (red) to low (blue). FDR, false discovery rate; NES, normalized enrichment score. (A–D) HALLMARK gene sets of (A) MYC_TARGETS_V1, (B) PI3K_AKT_MTOR_SIGNALING, (C) OXIDATIVE_PHOSPHORYLATION, and (D) G2M_CHECKPOINT are shown.

We next examined the effect of EGFR disruption on the gene expression in OSCC cells using cDNA microarray analysis. Differential gene expression analysis showed that 66 genes were downregulated (Table S1) and 162 genes were upregulated (Table S2) at transcriptional levels in the EGFR‐KO cells compared with those in the NT cells. GSEA with hallmark (h) gene sets showed that the oncogenic gene sets including Myc‐target and PI3K/Akt‐target genes were significantly downregulated in the EGFR‐KO cells (Figure 2A,B). In addition, oxidative phosphorylation and G2‐M checkpoint gene sets were inactivated in the EGFR‐KO cells (Figure 2C,D). These findings imply that the inactivation of oncogenic signaling and/or altered energy metabolism may be involved in the growth inhibition caused by EGFR deficiency.

### 3.3 VEGFR inhibitors preferentially suppress the proliferation of EGFR‐KO OSCC cells

**FIGURE 3:**
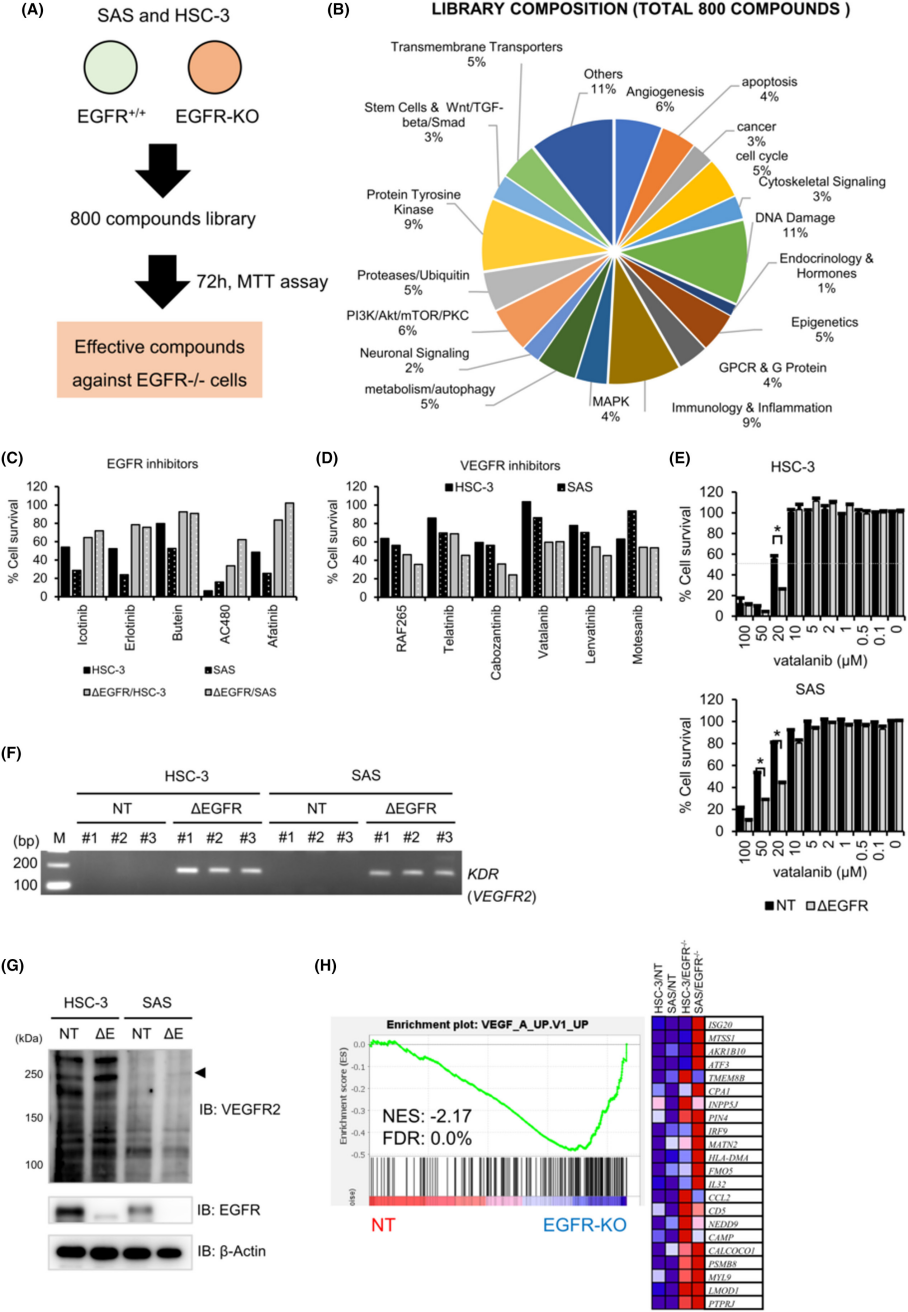
Drug sensitivity assay with a chemical compound library. (A) A schematic summary of drug screening assay. Cells (1 × 10^3^ cells/well) were incubated with each compound (total 800 compounds; final concentration at 5 μM) for 72 h. MTT assay was used for the measurement of the growth inhibitory effect. (B) Composition of the chemical compound library. (C, D) Representative assay results with EGFR TKIs (C) and VRGFR TKIs. (D) Percentage of cell survival is shown after normalization to the control cells (Mock treatment), whose survival is arbitrarily defined as 100%. (E) Effect of VEGFR inhibitor vatalanib on OSCC cell survival. The cells were incubated with the indicated concentration of vatalanib (100, 50, 20, 10, 5, 2, 1, 0.5, 0.1, and 0 μM) for 72 h. The results are shown as described above. An asterisk indicates a statistically significant difference between the NT and ΔEGFR cells (*N* = 3, *p* < 0.05). (F) RT‐PCR analysis. The HSC‐3 and SAS cell clones were incubated for 48 h. RT‐PCR analysis was performed at 30 cycles with specific primers for KDR/VEGFR2. A representative result of agarose gel electrophoresis was shown (*N* = 3). (G) Western blot analysis. Cell lysates were isolated from each cell and were applied to detect proteins with specific antibodies against VEGFR2, EGFR, and β‐Actin. β‐Actin was used as an internal control. (H) GSEA was conducted as described in Figure 2. All raw data were formatted and applied to the oncogenic signature gene sets (C6). The expression level is represented as a gradient from high (red) to low (blue). NT, non‐targeting; ΔE, EGFR‐deficient.

Since EGFR‐KO OSCC cells proliferated in vitro, but at a clearly slower rate than the NT cells under test, we examined how EGFR loss affected the sensitivity of OSCC cells to chemotherapeutic drugs. We utilized the customized chemical library consisting of 800 compounds including molecular‐specific inhibitors as well as FDA‐approved drugs (Figure 3A,B). MTT‐based drug screening assay showed similar but somewhat distinct patterns for sensitivities to each compound between the NT (*EGFR* ^*+/+*^) and the EGFR‐KO cells (Table S3). Treatment with erlotinib and afatinib, two EGFR inhibitors, caused predicted reductions in the survival rate in NT cells but essentially no cytotoxic effects in EGFR‐KO cells (Figure 3C; Table 1). In contrast, treatment with VEGFR inhibitors including vatalanib exhibited higher cytotoxic effects in EGFR‐KO cells than in NT cells (Figure 3D; Table 1). Furthermore, the MTT assay showed that the IC_50_ values of vatalanib in the EGFR‐KO cells were lower than those in the NT cells (HSC‐3, 22.2 μM vs. 16.1 μM; SAS, 53.2 μM vs. 17.6 μM in the NT and EGFR‐KO cells, respectively; Figure 3E). These results suggest that VEGFR signaling may play a pivotal role in the survival of *EGFR*‐deficient OSCC cells. Interestingly, RT‐PCR analysis revealed that *KDR*/*VEGFR2* gene expression was readily detectable in the EGFR‐KO HSC‐3 and SAS cells but not in the NT cells (Figure 3F). Moreover, Western blot analysis showed that the expression levels of VEGFR2 in the EGFR‐KO cells were stronger than those in the NT cells (Figure 3G). Furthermore, GSEA analysis with oncogenic signature gene sets showed that the VEGF_A_UP.V1_UP gene set was significantly activated in EGFR‐KO cells compared with that in NT cells (Figure 3H). These results suggest that VEGFR2‐mediated signaling might play an important role in the survival of OSCC cells.

**TABLE 1:** Representative result of MTT assay with chemical compounds.

Name	% cell survival						Target
	HSC‐3		SAS		Subtract		
	NT	ΔEGFR	NT	ΔEGFR	HSC‐3	SAS	
Effective chemical compounds against EGFR‐KO OSCC cells							
Cabozantinib (XL184)	59.4	36.0	56.3	24.3	23.5	32.1	VEGFR/c‐Met
MN 64	106.7	85.3	99.1	72.6	21.4	26.5	PARP
Vatalanib (PTK787) 2HCl	103.5	59.7	86.3	60.2	43.9	26.1	VEGFR
Lenvatinib (E7080)	77.8	54.6	70.1	45.2	23.2	24.9	VEGFR
Ki16198	104.6	80.9	92.9	68.6	23.7	24.2	LPA receptor
Less effective chemical compounds against EGFR‐KO OSCC cells							
Afatinib (BIBW2992)	48.5	83.6	25.5	102.2	−35.1	−76.7	EGFR/HER2
Erlotinib	52.4	78.5	24.0	75.7	−26.1	−51.8	EGFR
Carnosic acid	85.4	106.4	71.9	118.1	−21.1	−46.2	ROS
Carboprost	60.2	85.5	32.1	74.3	−25.2	−42.2	Others
(20S)‐Protopanaxatriol	33.5	77.0	40.6	71.0	−43.5	−30.5	Glucocorticoid receptor
Cyclocytidine HCl	9.3	31.5	11.3	40.2	−22.1	−28.8	DNA/RNA synthesis
AZ191	7.5	37.3	21.7	49.6	−29.8	−27.8	DYRK
Skp2 inhibitor C1 (SKPin C1)	37.4	93.6	42.1	68.8	−56.3	−26.7	CDK
Imatinib mesylate (STI571)	2.5	28.4	19.6	44.0	−25.9	−24.5	Bcr‐Abl, c‐Kit, PDGFR
Trifluridine	31.8	65.3	6.0	28.7	−33.5	−22.6	DNA/RNA synthesis
RKI‐1447	39.2	82.5	89.1	110.0	−43.3	−20.9	ROCK
Ripasudil hydrochloride dihydrate	50.2	90.6	86.1	106.2	−40.4	−20.1	ROCK

*Note*: The percentages of cell survival were calculated based on MTT assay, where HSC‐3 and SAS cells were incubated for 72 h in the presence or absence of each chemical compound at 5 μM.

### 3.4 Disruption of KDR/VEGFR2 suppresses the proliferation of OSCC cells

**FIGURE 4:**
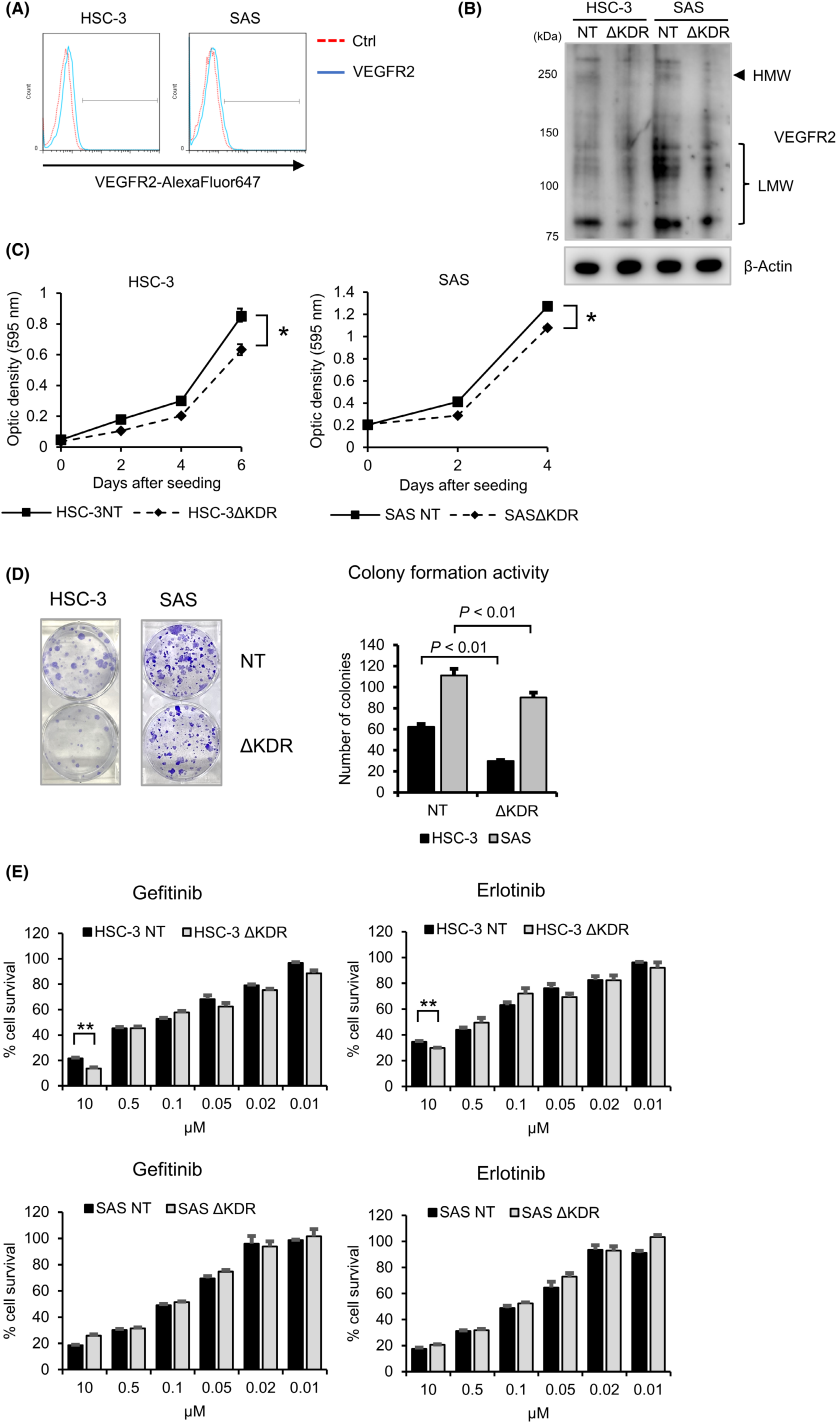
Effect of KDR disruption on cell growth of oral squamous cell carcinoma (OSCC) cells. (A) Flow cytometry analysis to detect the cell surface expression levels of VEGFR2 in HSC‐3 and SAS cells. (B) Western blot analysis. *KDR/VEGFR2* gene was disrupted with lentivirus‐mediated CRISPR‐Cas9 system. VEGFR2 protein expression was detected with anti‐VEGFR2 antibody (A5609, ABclonal). β‐Actin was used as an internal control. (C) OSCC cells (NT/HSC‐3, ΔKDR/HSC‐3, NT/SAS, and ΔKDR/SAS) were seeded into a 96‐well plate and were incubated for the indicated time (day‐0, day‐2, day‐4, and day‐6). The optic densities at each time points are shown. Solid line, NT cells; Dotted line, ΔKDR cells. (D) Colony formation assay. OSCC cells (200 cells/well in a 6‐well plate) were seeded and incubated for 10 days. The number of colonies is shown (*N* = 3, mean ± SE). (E) The effect of EGFR TKIs on cell survival of NT and ΔKDR OSCC cells. The cells were incubated with the indicated concentration (10, 0.5, 0.1, 0.05, 0.02, 0.01, and 0 μM) of gefitinib and erlotinib for 72 h. The relative percentages of cell survival are shown (*N* = 3, mean ± SE).

We investigated the role of KDR/VEGFR2 in the proliferation of OSCC cells. KDR‐KO OSCC cell lines were created for this purpose using the CRISPR/Cas9‐mediated lentivirus knockout technique (Figure 4A,B; Figure S2). Importantly, when compared to the NT cells, the *KDR/VEGFR2*‐KO HSC‐3 and SAS cells had considerably lower rates of cell survival and colony formation. (Figure 4C,D). Therefore, the sensitivities of the NT and *KDR/VEGFR2*‐KO cells toward anticancer drugs including EGFR kinase inhibitors gefitinib and erlotinib were assessed. MTT assay showed that the *KDR/VEGFR2*‐KO HSC‐3 cells were significantly more sensitive to the EGFR inhibitors gefitinib and erlotinib than the NT HSC‐3 cells, whereas the *KDR/VEGFR2* SAS cells and the NT cells showed no statistically significant differences in their sensitivities to the EGFR inhibitors (Figure 4E). These findings imply that VEGFR2‐mediated signaling, working in conjunction with EGFR signaling, may contribute to the proliferation of OSCC cells.

### 3.5 Combined EGFR and VEGFR2 inhibitors treatment cooperatively suppresses the proliferation of OSCC cells

**FIGURE 5:**
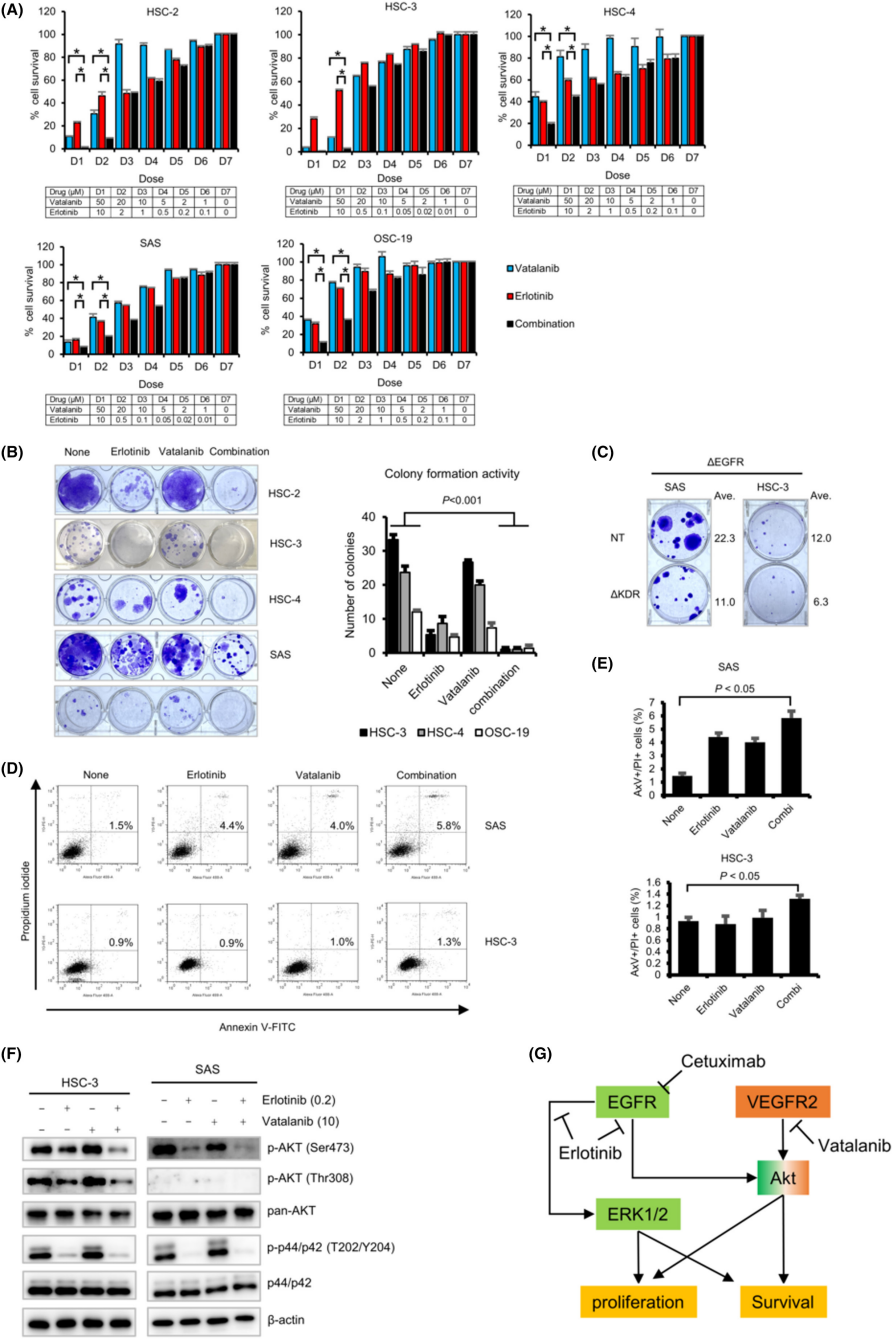
Effect of combined erlotinib‐vatalanib treatment on growth of OSCC cells. (A) OSCC cells (HSC‐2, HSC‐3, HSC‐4, SAS, and OSC‐19) cells were incubated with the indicated concentration of erlotinib (D1, D2, D3, D4, D5, D6, and D7 indicate 10, 2, 1, 0.5, 0.2, 0.1, 0 μM for HSC‐2, HSC‐4, OSC‐19 cells and for 10, 0.5, 0.11, 0.05, 0.02, 0.01, 0, respectively) and/or vatalanib (D1, D2, D3, D4, D5, D6, and D7 indicate 50, 20, 10, 5, 2, 1, and 0 μM) for 72 h. MTT assay was performed to determine the cell viability following treatment. The relative percentages of cell survival are shown as bar graphs with the dose information below each bar graph (*N* = 3, mean ± SE). Asterisks indicate a statistically significant difference between the cells subjected to combined erlotinib–vatalanib treatment and those subjected to either monotherapy (*N* = 3, *p* < 0.05). (B) Colony formation assay. OSCC cells were treated with erlotinib (0.2 μM) and/or vatalanib (10 μM) for 10–14 days. Representative results (left) and the number of colonies (right) are shown. (C) The effect of KDR disruption on clonogenic activity in the ΔEGFR HSC‐3 and SAS cells. The averages number of colonies are shown (*N* = 3). (D, E) The effect of combined erlotinib‐vatalanib treatment on apoptosis of OSCC cells. HSC‐3 and SAS cells were incubated with erlotinib (5 μM and 1 μM for HSC‐3 and SAS cells, respectively), vatalanib (10 μM), or combined erlotinib‐vatalanib for 48 h. The cells were stained with Annexin V (AxV)‐FITC and propidium iodide (PI). Representative results of AxV/PI assay are shown at the left. Bar graphs at the right show the percentages of AxV‐positive (AxV^+^) and PI‐positive (PI^+^) cells (*N* = 3, mean ± SE). (F) Western blot analysis showing the effect of combined erlotinib‐vatalanib treatment on the intracellular signaling pathway including Akt and p44/p42 in the OSCC cells. β‐Actin was used as an internal control. (G) The schematic summary depicting the possible crosstalk signaling related to cell survival in OSCC cells.

To assess the antiproliferative effect of EGFR with VEGFR inhibitors on OSCC, we tested the combined effect of vatalanib and erlotinib on the growth of OSCC cell lines in vitro. MTT assay showed that the combined treatment of vatalanib and erlotinib (vatalanib/erlotinib) caused higher suppression of OSCC growth as compared to vatalanib or erlotinib alone (Figure 5A). CI and DRI analyses for determining synergism and antagonism showed that vatalanib/erlotinib exerts a synergistic and/or additive effect on cell growth inhibition, with CI values ranging from 0.65 to 1.04 (at different effect levels from the IC_60_ to the IC_80_) in HSC‐2, from 0.917 to 1.04 (at those from the IC_50_ to the IC_70_) in HSC‐3, from 0.603 to 0.932 (at those from the IC_30_ to the IC_60_) in HSC‐4, from 0.664 to 0.807 (at those from the IC_30_ to the IC_60_) in OSC‐19, and from 0.698 to 1.034 (at those from the IC_30_ to the IC_60_) in SAS cells (where synergism, additivity, and antagonism are defined as CI <1, CI = 1, and CI >1, respectively; Figure S3). DRI analysis further indicated that vatalanib/erlotinib has the potential to reduce the doses of both vatalanib (ranging from 1.1‐fold to 10.6‐fold dose reduction) and erlotinib (ranging from 1.0‐fold to 35.4‐fold dose reduction) in the OSCC cell lines tested (Table S4). Additionally, compared to cells treated with either vatalanib or erlotinib alone, the number of colonies was much lower in the cells receiving combined therapy with the two drugs (Figure 5B). Similarly, colony formation assay showed that the VEGFR2/EGFR‐double KO cells had a relatively lower colony number than the NT/EGFR‐KO cells (Figure 5C). These results suggest that EGFR and VEGFR2 may cooperatively contribute to the proliferation of OSCC cells. Furthermore, AxV/PI‐double staining‐based FACS analysis revealed that compared to the untreated cells, the cells undergoing combined therapy with both the drugs showed a slight but significant increase in the apoptotic cell number (Figure 5D,E). Moreover, combined vatalanib and erlotinib treatment decreased the phosphorylation level of Akt (Ser473) much more than treatment with erlotinib or vatalanib alone (Figure 5F). In contrast, the phosphorylation level of p44/p42 MAPK in the cells receiving combined therapy with both medicines was similar to that in the cells treated with either vatalanib or erlotinib (Figure 5F). These results suggest that vatalanib may promote the tumor suppressive effect of erlotinib by cooperatively decreasing Akt activity (Figure 5G). In summary, the data presented here indicate the possibility that VEGFR2 signaling promotes the proliferation of OSCC cell cooperatively with EGFR signaling and targeting VEGFR2 might be a promising molecular target for treating patients with OSCC.

## 4 DISCUSSION

In this study, we examined the effect of EGFR disruption on the proliferation and drug sensitivity in the OSCC cell lines. CRISPR‐based genome editing system successfully generated the isogenic EGFR‐KO cell clones, in which proliferation was significantly suppressed compared with that of the control NT cells. Our drug screening assay revealed that while the EGFR‐KO cells were susceptible to VEGFR2 inhibitors, they were less sensitive to EGFR TKIs than the NT cells. Flow cytometry analysis detected the VEGFR2 expression in the OSCC cell lines HSC‐3 and SAS cells, where the disruption of *KDR/VEGFR2* gene significantly retarded the cell proliferation and colony forming activity. MTT assay revealed that combined EGFR and VEGFR2 treatment significantly suppressed the proliferation of OSCC cells concomitantly with a decrease in phosphorylation level of Akt.

*EGFR* gene, which encodes the EGFR tyrosine kinase, is abnormally amplified, rearranged, and mutated in a subset of cancers. Targeting abnormal EGFR is one of the major signal blockade strategies to treat patients with several cancers including OSCC. Our flow cytometry analysis showed high expression levels of EGFR in all the OSCC cell lines tested. EGFR is believed to play a pivotal role in the cellular growth of OSCC cells. As expected, disruption of *EGFR* led to obvious growth retardation in the EGFR‐KO cells compared with the growth of the NT cells. Indeed, GSEA showed that MYC target genes, PI3K‐Akt–mTOR signaling‐related genes, and oxidative phosphorylation were significantly inactivated in the EGFR‐KO cells. These results imply that EGFR is an indispensable receptor molecule for transducing the cellular proliferative response in OSCC.

The clinical outcome of cancer patients after receiving anti‐EGFR therapy is known to be diverse. One of the reasons for this is the differences in sensitivity against anti‐EGFR therapies, which are typically due to escaping of the blockade of survival signaling. In this study, we noticed that the EGFR‐KO cells still grew at a low proliferation rate. This result led us to hypothesize that despite the disruption of EGFR signaling, the OSCC cells may grow through an alternative viable signaling. Indeed, our drug screening analysis using a chemical library with 800 compounds showed that the EGFR‐KO cells were resistant to the EGFR inhibitors including gefitinib and erlotinib; however, they retained their sensitivity to the VEGFR inhibitors tested. Importantly, KDR/VEGFR2 expression and/or genes related to VEGF signaling clearly increased in the *EGFR*‐KO OSCC cells, indicating the possibility that the KDR/VEGFR2 signaling pathway may compensate the cell survival signaling in EGFR‐deficient OSCC cells.

*KDR/VEGFR2* gene, which encodes VEGFR2 tyrosine kinase, is involved in neovascularization to promote the supplementation of nutrients and oxygen for the proliferation of cancer cells.^33^ A variety of cancer cells have recently been found to express VEGFR2, in addition to the gene's significance in endothelial cells' angiogenesis.^34^, ^35^, ^36^, ^37^, ^38^, ^39^, ^40^, ^41^, ^42^, ^43^, ^44^ It has been observed that VEGFR2 enhances oncogenic signaling, hence promoting cell survival.^45^ VEGFR family members have also been reported to be expressed in OSCC tissues and a subset of cell lines.^46^, ^47^ Deng et al reported that VEGFR2 expression is inversely correlated with the overall survival of patients with OSCC.^48^ In this study, CRISPR‐mediated *KDR/VEGFR2* disruption suppressed the proliferation of HSC‐3 and SAS cells, suggesting that VEGFR2 may mediate the cell growth of OSCC.

Osude et al. recently discovered that VEGFR2 may be essential for EGFR TKIs to work in NSCLCs.^49^ They demonstrated that the erlotinib‐resistant NSCLC cell lines had higher expression of VEGFR2. In this study, we found that the VEGFR2 expression levels in the EGFR‐KO cells were relatively higher than those in the NT cells. Importantly, the sensitivity to EGFR TKIs gefitinib and erlotinib did not significantly differ between the NT and the KDR/VEGFR2‐KO OSCC cells. Although the involvement of other VEGFRs in the sensitivity to EGFR TKIs remains unclear, the result raises the possibility that other VEGF and VEGFR family members might also mediate the cell survival signaling and might be involved in the sensitivity to EGFR TKIs in OSCC cells.

The strategy to inhibit both EGFR and VEGFR signaling has been examined to improve the clinical outcome of patients with cancers including HNSCC.^50^, ^51^ Cohen et al showed that the combination of erlotinib and anti‐VEGF (a humanized monoclonal antibody bevacizumab) is well tolerated in recurrent or metastatic HNSCC, where a few patients achieved a favorable response.^51^ In this study, the co‐treatment of erlotinib with the VEGFR2 inhibitor vatalanib prevented OSCC cells from proliferating and forming colonies, which was accompanied by an increase in the number of apoptotic cells. Subsequently, we found that the phosphorylation level of Akt (Ser473) was lower in the cells that received combined erlotinib‐vatalanib treatment than in the cells receiving either monotherapy. These findings indicate that EGFR and VEGFR inhibitions together limit OSCC cell proliferation, at least in part, via weakening the PI3K‐Akt signaling pathway. Since targeting VEGFR signaling may reduce tumor neovascularization in vivo, VEGFR inhibition may suppress tumor growth both directly and indirectly in OSCC. Further studies with in vivo xenograft experiments are warranted to uncover the role of VEGFR signaling in tumor development of OSCC.

Targeting EGFR has currently become one of the major signal blockade strategies to treat patients with several cancers including OSCC. Although anti‐EGFR antibody cetuximab with other chemotherapies and/or radiotherapy has been used to treat patients with OSCC, the clinical outcome and/or the quality of life of the patients remains low due to drug resistance. Our research investigated drug sensitivity of OSCC cells when EGFR signaling was inhibited, and dual inhibition of EGFR and VEGFR might be a viable cutting‐edge therapeutic approach to enhance the clinical result of OSCC patients.

## AUTHOR CONTRIBUTIONS

**Maho Onda:** Data curation (lead); formal analysis (equal); investigation (lead). **Akinobu Ota:** Conceptualization (lead); data curation (equal); formal analysis (equal); funding acquisition (lead); writing – original draft (equal). **Kunihiro Ito:** Conceptualization (equal); data curation (equal); investigation (equal); project administration (equal). **Takayuki Ono:** Investigation (equal); resources (equal); supervision (equal). **Sivasundaram Karnan:** Formal analysis (equal); investigation (equal); methodology (equal); visualization (equal). **Mikako Kato:** Data curation (equal); formal analysis (equal); methodology (equal). **Sayuri Kondo:** Methodology (equal); supervision (equal). **Akifumi Furuhashi:** Supervision (equal); validation (equal). **Tomio Hayashi:** Resources (equal); supervision (equal). **Yoshitaka Hosokawa:** Conceptualization (equal); project administration (equal); supervision (equal); writing – review and editing (equal). **Yoshiaki Kazaoka:** Conceptualization (equal); funding acquisition (equal); project administration (equal); supervision (equal); validation (equal).

## FUNDING INFORMATION

This work was partly supported by Grants‐in‐Aid for Scientific Research (KAKENHI) from the Japan Society for the Promotion of Science (18K08342 to A.O., 22K08294 to A.O. and Y.H., 22K08985 to S.K., 21K08426 to A.O. and S.K.) and the Research Grant from the Hori Science and Arts Foundation (HF03‐2021‐0012 to A.O.).

## CONFLICT OF INTEREST STATEMENT

The authors declare no competing interests.

## Supporting information


Figure S1.

Figure S2.

Figure S3.

Table S1.

Table S2.

Table S3.

Table S4.

## Data Availability

The data that support the findings of this study are openly available in GEO database at NCBI at [https://www.ncbi.nlm.nih.gov/geo/query/acc.cgi?acc=GSE222629], reference number [GSE222629].
