# Supplementary figures and images for: Inhibition of VEGFR2 and EGFR signaling cooperatively suppresses the proliferation of oral squamous cell carcinoma
Source: Cancer Med. 2023 Jun 21;12(15):16416–30. doi: 10.1002/cam4.6282 (PMC10469792; doi:10.1002/cam4.6282)

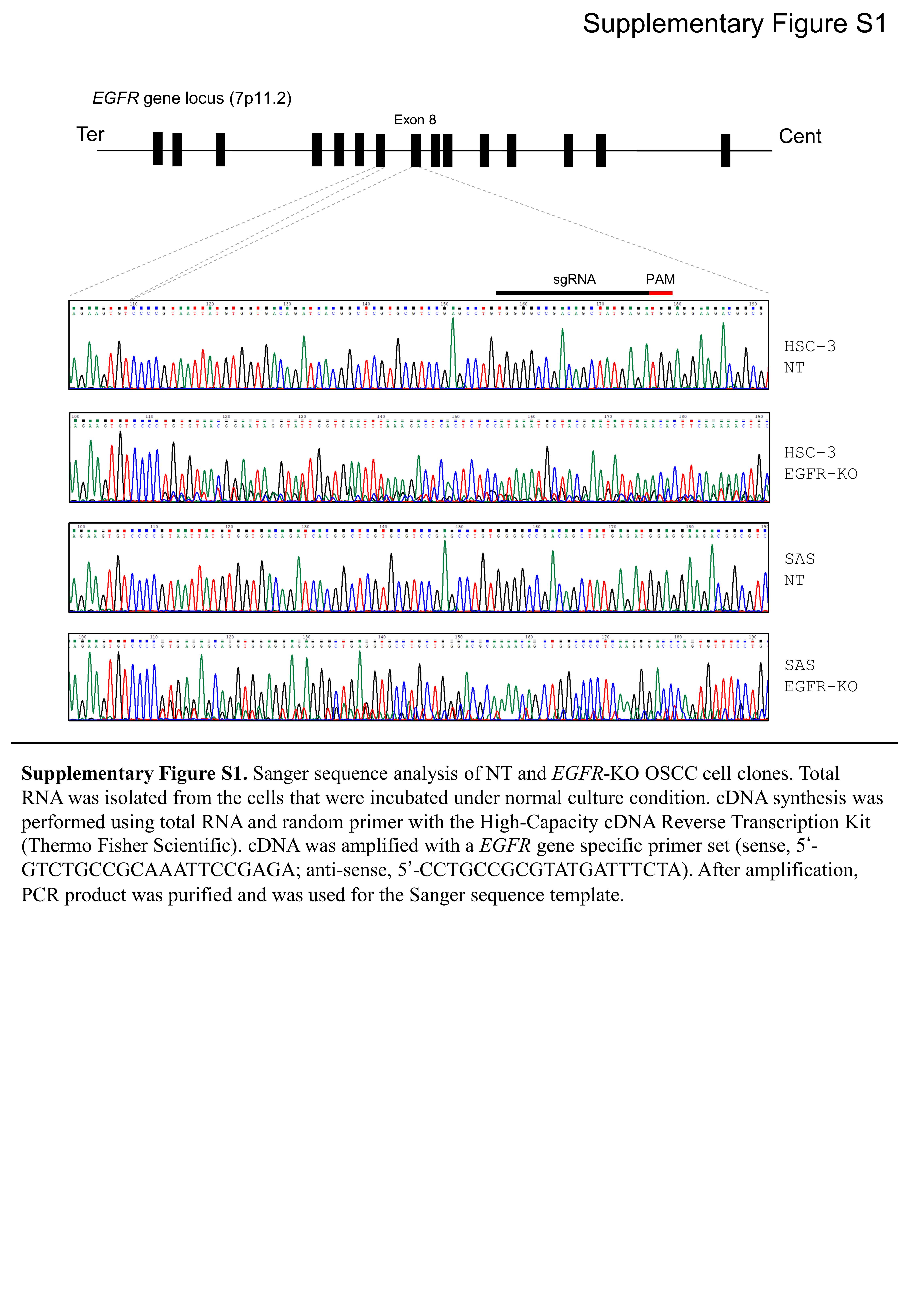

Supplement: Supplementary file 1 — Figure S1. [file CAM4-12-16416-s002.tif]

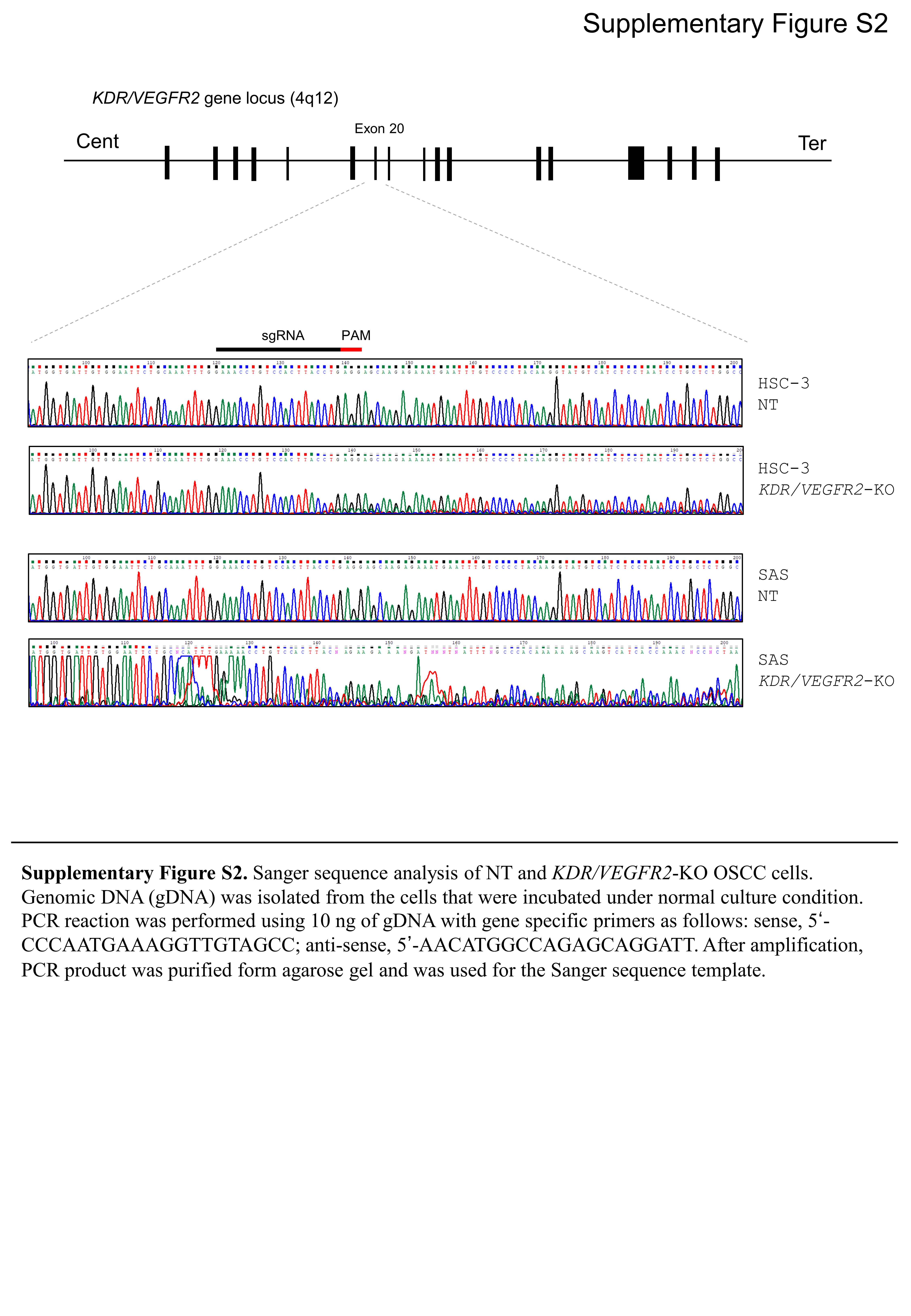

Supplement: Supplementary file 2 — Figure S2. [file CAM4-12-16416-s003.tif]

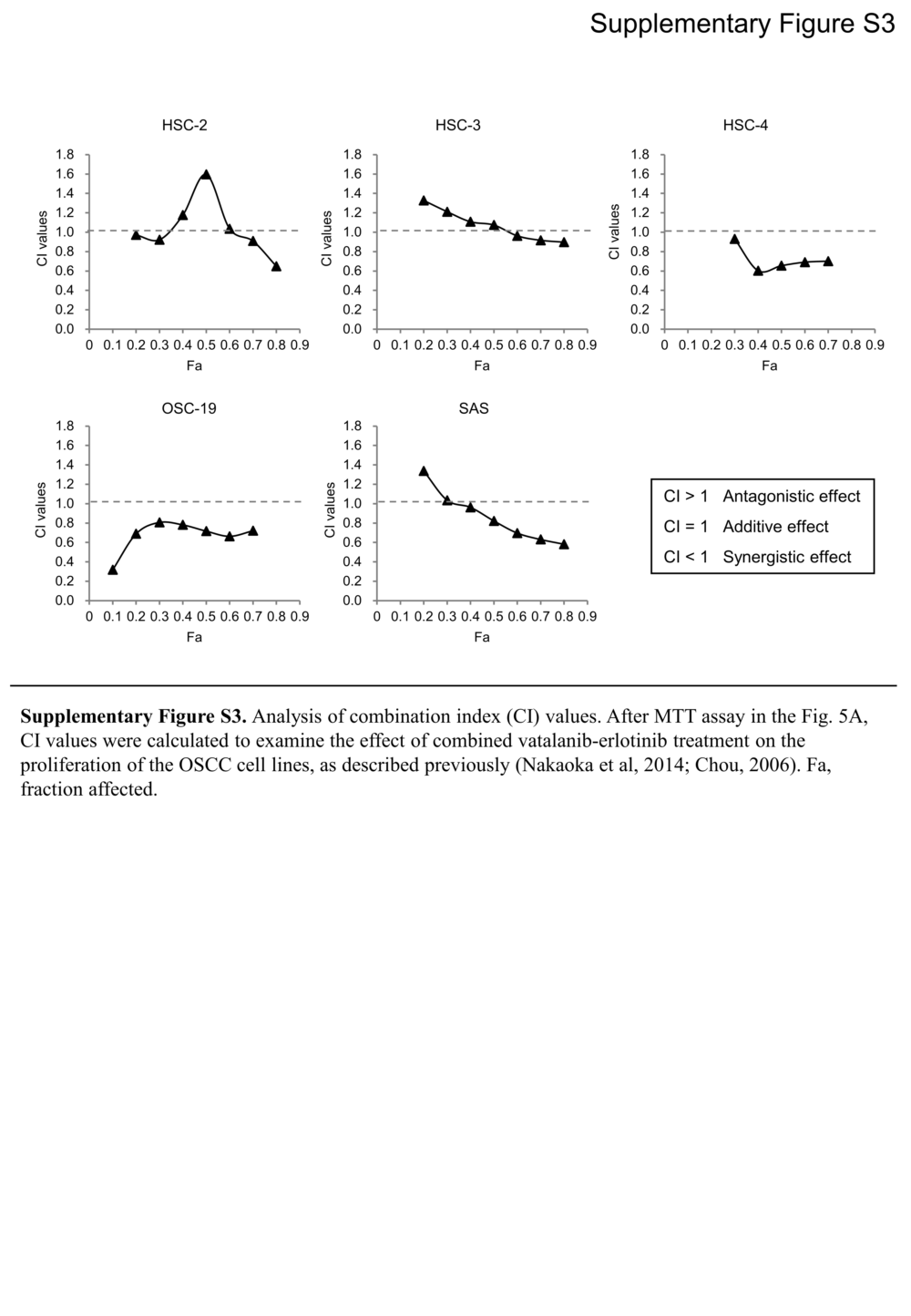

Supplement: Supplementary file 3 — Figure S3. [file CAM4-12-16416-s006.tif]
